# Reconstruction of Hand Dorsum Defect Using Double Perforators-Based Anterior Interosseous Artery Island Flap: A Case Report and Description of a New Anterior Interosseous Artery Perforator

**DOI:** 10.1055/a-2067-5403

**Published:** 2023-08-02

**Authors:** Inho Kang, Hyun Rok Lee, Gyu Yong Jung, Joon Ho Lee

**Affiliations:** 1 Department of Plastic and Reconstructive Surgery, College of Medicine, Dongguk University, Gyeongsangbuk-do, Republic of Korea; 2 Department of Plastic and Reconstructive Surgery, Saeson Hospital, Daejeon, Republic of Korea

**Keywords:** anterior interosseous artery, anterior interosseous flap, perforator flap, posterior interosseous artery flap, radial artery perforator flap

## Abstract

The anterior interosseous artery (AIA) perforator flap is not commonly used in hand dorsum reconstruction compared with alternatives. However, it is a versatile flap with several advantages. Literature on the AIA perforator flap is based on the dorsal septocutaneous branch (DSB), which branches from the AIA and passes through fascia between the extensor pollicis longus (EPL) and extensor pollicis brevis muscles. In the described case, the authors reconstructed a hand dorsum defect in a 78-year-old man using an AIA perforator flap with double perforators supplied by the DSB and a new perforator branching from the distal than DSB. No complication was encountered, and the flap survived completely. A retrospective computed tomography review revealed the presence of the new perforator in 14 of 21 patients. Two types of new perforator were observed. One passed through the ulnar side of the extensor indicis proprius (EIP) muscle and penetrated fascia between the extensor digitorum minimi and extensor digitorum communis tendons, whereas the other passed between the EPL and EIP muscles. This report describes the anatomical location and clinical application of the new AIA perforators. The double perforators-based AIA flap provides a straightforward, reliable means of reconstructing hand dorsum defects.

## Introduction

The dorsum of the hand is composed of thin skin and subcutaneous tissue, 1 which makes this region vulnerable to injury. When such injuries result in tendon or bone exposure, skin grafting is not possible, and the defect must be covered with a flap such as a local, distant, or free flap. Depending on underlying disease and general condition, local flaps present lower risks than distant or free flaps. The options available for hand dorsum local flaps include the radial artery perforator (RAP) flap, radial forearm flap (RFF), posterior interosseous artery (PIA) flap, and ulnar artery perforator (UAP) flap. 1 2 3 4 5 6 Perforators also branch from the anterior interosseous artery (AIA) at the dorsal wrist, but it is not commonly used. However, depending on the situation, an AIA perforator flap can be a good option for hand dorsum defect coverage. In this paper, we performed a double perforators-based AIA pedicled flap using a new AIA perforator that has not been described so far. In addition, by using double perforators, complications were reduced compared with the AIA pedicled flap performed with one perforator. In addition, we retrospectively reviewed computed tomography (CT) angiography findings to determine the prevalence and location of the new second perforator. Here, we describe a case in which a hand dorsum defect was covered with a double perforators-based AIA island flap. Written informed consent was obtained from the patient for the publication and use of his case details and photographs.

## Case

**Fig. 1:**
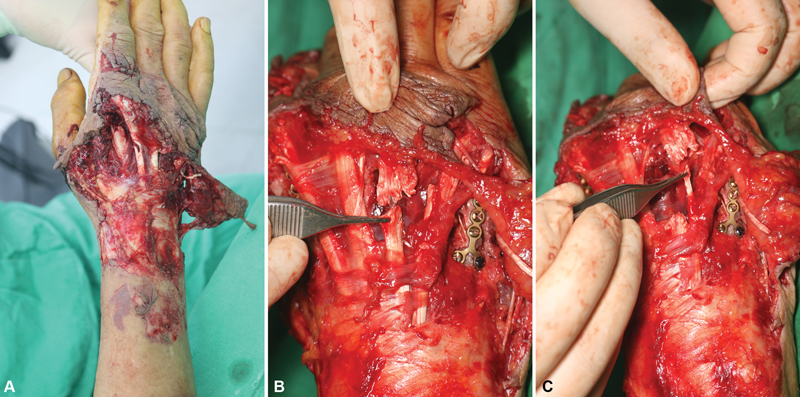
Initial photograph. ( **A** ) Large degloving wound of the dorsum of the Rt. hand and distal forearm. ( **B** ) Complete rupture of the Rt. third and fourth EDC tendons. ( **C** ) Complete rupture of Rt. EDM tendon. EDC, extensor digitorum communis; EDM, extensor digitorum minimi; Rt, right.

**Fig. 2:**
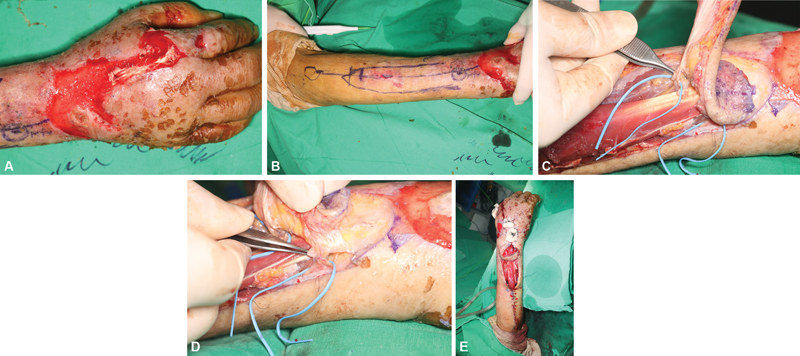
( **A** ) Photograph taken 2 weeks after the first procedure showing exposure of the second EDC tendon due to a soft tissue defect in the hand dorsum. ( **B** ) Double perforators-based AIA propeller flap design. ( **C** ) Photographs showing a septocutaneous perforator (DSB) passing between the extensor pollicis longus and extensor pollicis brevis muscles. ( **D** ) A septocutaneous perforator passing through the ulnar side of the extensor indicis proprius muscle. ( **E** ) Flap inset. The flap was rotated clockwise by 120 degrees. AIA, anterior interosseous artery; DSB, dorsal septocutaneous branch; EDC, extensor digitorum communis.

**Fig. 3:**
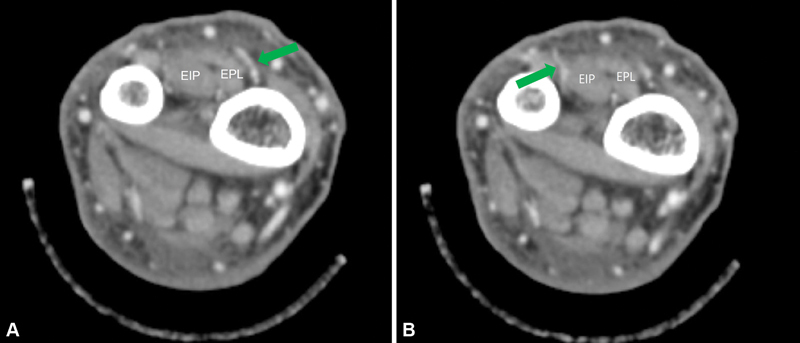
Preoperative upper extremity CT angiography. ( **A** ) A septocutaneous perforator (DSB) passed between the extensor pollicis longus and extensor pollicis brevis at 52 mm proximal to the ulnar styloid process. ( **B** ) A septocutaneous perforator passed through the ulnar side of the extensor indicis proprius muscle at 32 mm proximal in the ulnar styloid process. CT, computed tomography; DSB, dorsal septocutaneous branch.

A 78-year-old man with high blood pressure and diabetes presented at our emergency department after being involved in a traffic accident. Physical examination revealed a degloving wound of the right hand and distal forearm with complete rupture of multiple tendons ( Fig. 1 ). In addition, hand X-ray and CT findings revealed fractures of the second and fifth metacarpal bone. The metacarpal fractures were treated by open reduction and internal fixation, and tenorrhaphy was performed for the tendon injuries. To treat the degloving wound of the hand dorsum, we decided to perform primary repair and monitor progress. Two weeks after primary repair, skin of the degloving wound was partially necrotized, and the second extensor digitorum communis (EDC) tendon was exposed ( Fig. 2A ). The size of the defect was 10 cm × 3 cm, and thus, flap surgery was required to achieve coverage. Furthermore, because the patient was elderly and in poor general condition, reconstruction was attempted using a local flap rather than a free flap. The flap was designed with the aid of CT angiography, which showed that anastomosis between the AIA and the PIA was damaged, and thus, the PIA flap was excluded because it requires this anastomosis as a pedicle. In addition, the RAP flap was excluded due to poor skin quality because there was a burn scar on the anterior side of the forearm. The UAP flap was also excluded because the defect was located on the radial side of the hand dorsum. Therefore, we decided on reconstruction using the AIA perforator flap and planned to cover the donor defect by primary closure and skin grafting. CT angiography revealed the presence of two AIA perforators ( Fig. 3A and B ); one located 52 mm proximal from the ulnar styloid process and the other 32 mm proximal from ulnar styloid process. So, we decided to reconstruct using an AIA perforator propeller flap with double perforators.

**Fig. 4:**
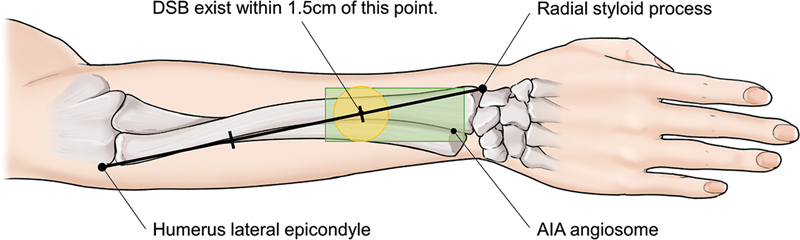
Schematic diagram. AIA angiosome and drawing for finding DSB. DSB was found that if a straight line is drawn from the radial styloid process to the humerus lateral epicondyle after pronation of the forearm, the DSB exists within 1.5 cm of the point dividing the middle and distal thirds of the line. 13 AIA, anterior interosseous artery; DSB, dorsal septocutaneous branch.

**Fig. 5:**
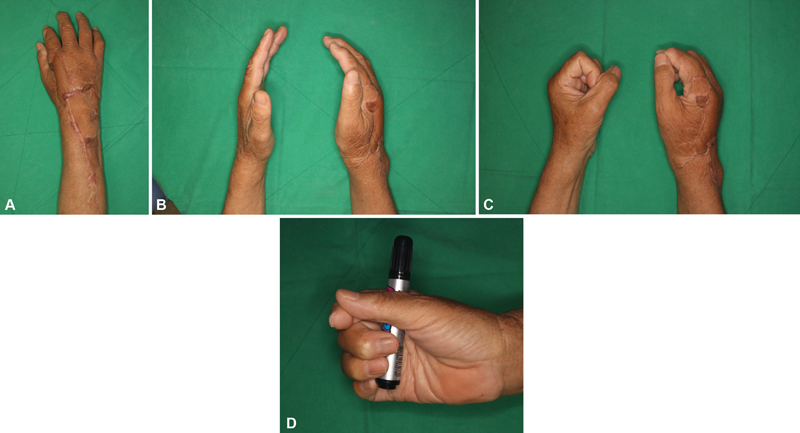
Eight-month postoperative photographs. ( **A** ) No complication was encountered. ( **B** ) Active full extension of hand. ( **C** ) Active full flexion of hand. There were limitations in the flexion and extension functions. ( **D** ) The patient recovered some amount of grip function.

An anesthesiologist administered brachial plexus block, and the flap was designed. Briefly, the forearm was pronated, and a straight line was drawn from the styloid process of the radius to the lateral epicondyle of the humerus. On dividing this line into three equal parts, an AIA perforator may be found at the point between the middle and distal thirds ( Fig. 4 ). 7 Doppler ultrasonography was used to locate two perforators, which were then marked. A propeller flap was then designed to cover the hand dorsum using the perforators as pivot point ( Fig. 2B ). A tourniquet was inflated, and an incision was made on the ulnar side of the flap. Subfascial dissection was then performed, and two septocutaneous perforators were found ( Fig. 2C and D ). The propeller flap using double perforators was elevated and rotated clockwise by 120 degrees to inset the defect ( Fig. 2E ). The size of the flap was 15 cm × 3 cm. The donor site was closed by primary repair using a split-thickness skin graft. No complication was encountered, and the flap survived completely ( Fig. 5A ). Postoperative rehabilitation was started 2 weeks after flap placement. However, because of the pain, the patient was not active in rehabilitation. Because of this, there were limitations in the flexion and extension functions ( Fig. 5B and C ). It was particularly severe in the index finger. However, the patient said that a hand function was restored sufficiently to enable daily activities ( Fig. 5D ).

## Discussion

Hand dorsum defects can be covered in several ways depending on defect size. For small defects, random pattern local flaps and skin grafts can be used, but when a tendon or bone is exposed defects must be covered with a flap. For medium to large defects, reconstruction options include a local pedicle flap, a distant flap, or a free flap, and depending on underlying disease and general condition, a local pedicle flap has a lower risk than a distant or free flap. In the described case, reconstruction was attempted using an AIA perforator-based local flap rather than a free flap, because the patient was an elderly trauma patient in poor general condition.

Local pedicle flaps for the hand dorsum include the RAP flap, RFF, PIA flap, AIA perforator flap, and UAP flap. 5 For radial side defects, RAP flap and RFF are mainly used. 5 PIA flap and AIA perforator flap can be applied for central defects, and ulnar defects are covered with a UAP flap. 5 The RAP flap is based on the RAP as the pedicle and is an excellent option for covering the hand dorsum without damaging the main artery and mainly used to cover small to medium defects on the radial side of the hand dorsum. 3 5 Unfortunately, our patient had a burn scar on the volar side of the distal forearm, and thus, this site could not be used as the donor site for an RAP flap due to poor skin quality. The RFF is based on the radial artery as pedicle. This flap has the advantages of being easily raised and reliable 3 5 and suitable for hand dorsum defect reconstruction because it provides thin, pliable skin. 3 5 However, it requires sacrifice of the main artery of the upper extremity, 3 5 and thus might impair circulation in an already injured hand. On the other hand, the PIA flap is based on the PIA, and when performed with a local pedicled flap, anastomosis of PIA and AIA is used as the pedicle. 3 5 8 However, anatomical variations of the PIA are common, and sometimes the PIA is absent. 3 5 8 In other cases, no anastomosis occurs between the PIA and AIA. These common anatomical variations can make flap dissection difficult. 8 Also, the flap pedicle is shorter than the AIA flap. 9 In the described case, a large defect was present in the hand dorsum and distal forearm, and CT angiography revealed no anastomosis between the PIA and AIA, and thus, the PIA flap was excluded.

Taylor and Palmer first introduced the angiosome concept in 1987. 10 In this paper, the AIA angiosome was introduced as the 39th angiosome. Inoue and Taylor described the forearm angiosome in detail in 1996. 11 In this paper, AIA perforators were described for the first time. Shibata and Ogishyo first described the AIA perforator flap in 1996, 12 and since, all reports on AIA flaps involve the use of the dorsal septocutaneous branch (DSB) of the AIA as pedicle. 6 7 9 12 13 The DSB passes through the radial side of the extensor pollicis longus (EPL) and then courses along the ulnar side of the radius and passes through fascia between the extensor pollicis brevis (EPB) and EPL ( Fig. 3A ). 7 9 12 13 Reported DSB thicknesses range from 0.9 to 1.6 mm. 6 7 9 12 13 The DSB has been reported to be present in almost everyone and always with vena comitans. 6 9 12 13 The location of the DSB has been variously described in the literature. In one study, the DSB was located 3 to 4 cm proximally and 0.5 to 1 cm ulnarly from Lister's tubercle. 9 In another study, it was found that if a straight line is drawn from the radial styloid process to the humerus lateral epicondyle after pronation of the forearm, the DSB exists within 1.5 cm of the point dividing the middle and distal thirds of the line ( Fig. 4 ). 13 It was also reported that the DSB is located 44 mm proximally from the ulnar styloid process. 6 The main advantage of the AIA flap is that its use does not involve sacrifice of the main artery, and its disadvantage is that it leaves a visible scar on the posterior forearm.

In this case, we used a double perforators-based AIA propeller flap using the DSB and another AIA perforator. The presence of these two perforators was visualized by CT angiography and Doppler ultrasonography. More specifically, the DSB (a septocutaneous perforator) branched from the AIA at 52 mm proximal to the ulnar styloid process ( Fig. 3A ), passed through the radial side of the EPL muscle, penetrated fascia between the EPL and EPB. The other septocutaneous perforator has not been previously described and branched from the AIA at 32 mm proximal to the ulnar styloid process ( Fig. 3B ). After branching from the AIA, this perforator passed through the ulnar side of the extensor indicis proprius (EIP) muscle and penetrated fascia between the EDC and EDM tendons. We performed local pedicled propeller flap surgery using these two perforators and successfully reconstructed a hand dorsum defect. Previously, we had performed three AIA perforator-based island flap surgeries based on DSB as a single perforator. All flaps survived, but congestion occurred for a few days after surgery, and a flap salvage procedure was required in all cases. It was also reported in a clinical study that congestion occurred for some days after single perforator-based AIA island flap surgery. 6 On the other hand, the described double perforators-based flap was successful in our patient, who displayed excellent circulation without congestion and no wound complication.

**Fig. 6:**
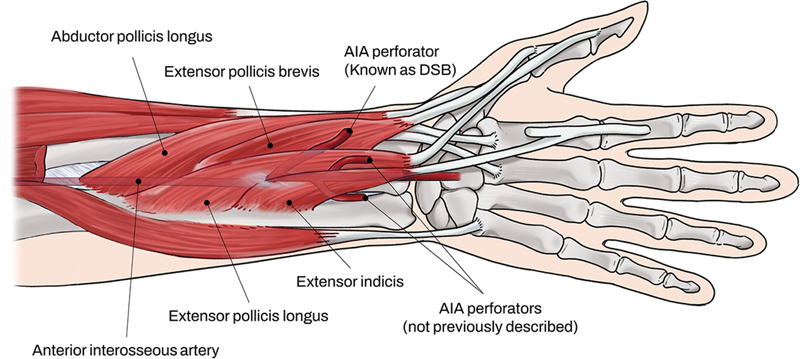
Schematic diagram. The anatomical location of AIA perforators. AIA, anterior interosseous artery.

**Fig. 7:**
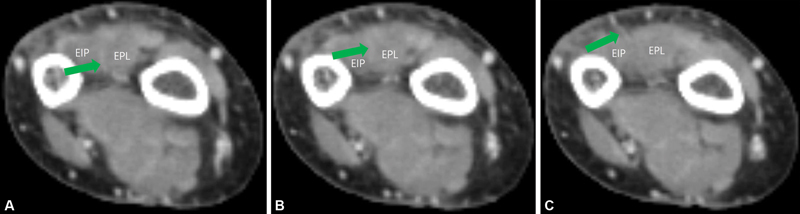
( **A–C** ) A septocutaneous perforator was branched from the AIA and passed between the EIP and EPL muscles. AIA, anterior interosseous artery; EIP, extensor indicis proprius; EPL, extensor pollicis longus.

We retrospectively reviewed CT angiography findings of all 21 patients that underwent upper extremity CT angiography at our hospital from 2012 to 2022 to determine the prevalence of the new second perforator. The exclusion criteria applied were: (1) the presence of peripheral arterial occlusive disease, (2) a CT angiography slice thickness of >2 mm, and (3) a CT resolution too low to allow adequate perforator visualization. Mean patient age was 61.0 ± 19.4, and there were 15 males and 6 females. Two types of new second perforators were detected and both were septocutaneous ( Fig. 6 ). One had the same form as in the described case, that is, the perforator passed through the ulnar side of the EIP muscle and penetrated the fascia between the EDM and EDC tendons ( Fig. 3B ), whereas the other type passed between EPL and EIP muscles ( Fig. 7 ). In the 21 patients, the perforator passed through the ulnar side of the EIP muscle in six patients and between the EPL and EIP in eight. No second new perforator was found in seven patients. There were no cases in which two types of new perforators coexisted. A new perforator branches from the AIA were found at 40.3 ± 6.9 mm proximal to the ulnar styloid process. Notably, the ability of CT angiography to image perforators is limited. We used a CT slice thickness of 1 to 2 mm, which is larger than perforator thicknesses. Additional study is required to obtain more detailed information about this perforator.

The double perforators-based AIA island flap has many advantages. Most importantly, it does not require sacrifice of the main artery, which is associated with complications such as cold intolerance, hand stiffness, and varying degrees of sensory loss. 3 5 8 Another advantage is that two septocutaneous perforators are used. Both perforators are easily found by dissection along the subfascial layer, which reduces operation time. In addition, if one perforator is injured intraoperatively, the flap can be attempted using the other perforator. Furthermore, the circulation is significantly improved, and risks of flap complications, such as congestion, are reduced when two perforators are used. In addition, we believe that the second new AIA perforator could be utilized in different ways, even in free flaps by means of turbocharge or supercharge. The disadvantages of this flap are that it leaves a scar on the posterior forearm and primary closure of the donor site of the AIA flap is difficult. Thus, a skin graft is required, which may leave a large visible scar on the posterior forearm.

The limitation of this report is that only one case of surgery was performed clinically. In addition, we reviewed the AIA perforator with CT angiography, but the number of patients was small, only 21 patients. We used a CT slice thickness of 1 to 2 mm, which is larger than perforator thicknesses. Therefore, we think that there are many perforators that did not appear on CT angiography. Because of these limitations, further studies are needed.

AIA perforator flap is less mentioned in textbooks or review articles than other alternatives. However, considering its advantages, we recommend it be considered an essential option for hand dorsum reconstruction. In particular, when performed using double perforators, the procedure is straightforward, and the success rate is high. Therefore, we argue that the AIA flap using double perforators is a good option for hand dorsum reconstruction.
